# Examining the Light Heart Mobile Device App for Assessing Human Pulse Interval and Heart Rate Variability: Validation Study

**DOI:** 10.2196/56921

**Published:** 2024-08-20

**Authors:** Stephen A Klassen, Jesica Jabbar, Jenna Osborne, Nathaniel J Iannarelli, Emerson S Kirby, Deborah D O'Leary, Sean Locke

**Affiliations:** 1 Faculty of Applied Health Sciences Brock University St. Catharines, ON Canada; 2 Light Heart Kelowna, BC Canada

**Keywords:** pulse interval, mobile app, validation, mHealth, mHealth app, app mobile device, mobile device app, pulse, heart, heart rate, validation study, biomarker, psychological, physiological, pulse rate, young adults, youth, linear correlation, heart rate variability, examining, examine, validity, psychological health, physiological health, interval data, mobile phone

## Abstract

**Background:**

Pulse interval is a biomarker of psychological and physiological health. Pulse interval can now be assessed using mobile phone apps, which expands researchers’ ability to assess pulse interval in the real world. Prior to implementation, measurement accuracy should be established.

**Objective:**

This investigation evaluated the validity of the Light Heart mobile app to measure pulse interval and pulse rate variability in healthy young adults.

**Methods:**

Validity was assessed by comparing the pulse interval and SD of normal pulse intervals obtained by Light Heart to the gold standard, electrocardiogram (ECG), in 14 young healthy individuals (mean age 24, SD 5 years; n=9, 64% female) in a seated posture.

**Results:**

Mean pulse interval (Light Heart: 859, SD 113 ms; ECG: 857, SD 112 ms) demonstrated a strong positive linear correlation (*r*=0.99; *P*<.001) and strong agreement (intraclass correlation coefficient=1.00, 95% CI 0.99-1.00) between techniques. The Bland-Altman plot demonstrated good agreement for the mean pulse interval measured with Light Heart and ECG with evidence of fixed bias (–1.56, SD 1.86; 95% CI –5.2 to 2.1 ms), suggesting that Light Heart overestimates pulse interval by a small margin. When Bland-Altman plots were constructed for each participant’s beat-by-beat pulse interval data, all participants demonstrated strong agreement between Light Heart and ECG with no evidence of fixed bias between measures. Heart rate variability, assessed by SD of normal pulse intervals, demonstrated strong agreement between techniques (Light Heart: mean 73, SD 23 ms; ECG: mean 73, SD 22 ms; *r=*0.99; *P*<.001; intraclass correlation coefficient=0.99, 95% CI 0.97-1.00).

**Conclusions:**

This study provides evidence to suggest that the Light Heart mobile app provides valid measures of pulse interval and heart rate variability in healthy young adults.

## Introduction

Autonomic regulation of cardiac function represents a key mechanism for homeostatic maintenance in humans. As a result of autonomic regulation, heart rate demonstrates strong beat-to-beat variability, described commonly as heart rate variability (HRV). Given that the autonomic nervous system is comprised of a network of nuclei distributed throughout the brain and brainstem, HRV provides an avenue to explore the brain-heart connection in humans [1,2].

HRV represents a biomarker of health status and acute physiological and mental health [3]. HRV declines strongly with aging and is reduced in many chronic cardiovascular [4], metabolic [5], and neurological diseases [6]. Thus, HRV represents a strong predictive biomarker for all-cause mortality, cardiovascular outcomes, depression, and dementia [7]. HRV has also been characterized as an index of emotion regulation [8,9].

HRV can be measured in time and frequency domains using the R-R intervals provided by electrocardiograms (ECGs) [10]. However, ECG-based measures of HRV can be prohibitive due to the requirement for technical equipment and specialized expertise to support ECG collection and HRV analysis. Accordingly, to extend HRV measures beyond laboratory and clinical environments, methods have been developed that rely on photoplethysmography (PPG) to measure pulse interval from the pulsatile changes in microcirculatory blood volume in an accessible anatomical location such as a finger [11].

The recent uptick in wearable technologies capable of performing photoplethysmographic measures of pulse interval has mitigated some of the barriers to population-level HRV assessment [12]. However, a limited proportion of households in low-income nations report using wearable technology [13]. Conversely, smartphone use is high (85%+) among residents of high-income nations such as Canada and the United States [14]. Thus, mobile phones equipped with software capable of performing photoplethysmographic measures of HRV will support the scaling of this measure to the population level. In this regard, Light Heart is a novel mobile app that uses the camera and flash of a mobile device to obtain continuous measures of pulse interval from an individual’s fingertip using PPG. To date, the validity of pulse interval and HRV indices measured by Light Heart has not been investigated. Like other HRV apps [15,16], measurement accuracy should be established prior to releasing an app to the public.

Many commercially available apps that use PPG to measure pulse interval provide a single metric of stress but do not provide researchers with the raw data (eg, Fitbit and Apple). These single metrics conceal beat-by-beat data and prevent researchers from examining raw data points that may skew aggregate indices or be used to calculate several time- and frequency-domain indices of HRV. As a result, researchers may be limited in their ability to obtain beat-by-beat pulse interval data in real-world studies. Furthermore, there is a reported lack of rigor in evaluating apps that measure heart rate or HRV [17]. The Light Heart app was developed to measure pulse intervals outside the laboratory. The metrics obtained by new research tools require validation to ensure accuracy and consistency prior to use in applied research settings. Validation is defined as the process of gathering evidence to suggest a tool is able to accurately measure what it proports to measure. [18]. An initial step in the process of validation is to demonstrate that an assessment is concurrently valid and provide accurate estimates against a gold standard like an ECG [19]. Therefore, this study aimed to investigate the validity of the Light Heart mobile app to quantify pulse interval and HRV using ECG as the gold-standard reference in a cohort of healthy young individuals. This study tested the hypothesis that pulse interval and HRV would demonstrate strong agreement between Light Heart and ECG.

## Methods

### Ethical Considerations

This study received approval from Brock University’s Health Science Research Ethics Board (#21-118) and conforms to the Declaration of Helsinki, except for registration in a database. Each participant provided informed written consent after receiving detailed written and verbal explanations of study procedures. Study data are deidentified and stored on an institutional secure server. Participants were not remunerated for participation.

### Participants

This study tested 14 young healthy, nonsmoking adults (n=9, 64% female and n=5, 36% male; mean age 24, SD 5 years; mean height 169, SD 7 cm; mean weight 68, SD 10 kg; n=11, 79% White; n=1, 7% Hispanic or Latino; and n=2, 14% Southeast Asian). Participants were normotensive and free from cardiovascular and other diseases and major risk factors. Participants were not currently taking medications known to affect autonomic function, heart rate, or blood pressure. Both oral contraceptive–using and –nonusing female individuals were included. This study did not control for the menstrual phase among female participants. Based on previous studies [20], a priori sample size calculation revealed a target sample size of 14 based on detecting a minimum correlation of *r*=0.7, with α=.01 and power (1 – β) = 0.8.

### Experimental Protocol

Participants arrived at the Human Hemodynamics Laboratory at Brock University after at least a 4-hour fast and abstaining from alcohol, nicotine, caffeine, cannabis, and vigorous physical activity for at least 12 hours prior to the study session. Height (STAT 7X, Ellard Instrumentation Ltd) and body mass (BWB-800S, Tanita Corporation) were measured.

Participants were instrumented in the supine posture. Following at least 10 minutes of rest, participants were familiarized with the mobile device featuring the Light Heart app providing the pulse interval recordings. Participants were asked to perform at least a 60-second recording with the mobile device to ensure familiarization and for investigators to verify optimal pulse recordings. Participants then transitioned to a seated position where they assumed a normal seated posture with legs uncrossed and feet positioned on the floor. The experimental protocol consisted of a 5-minute seated condition with simultaneous recordings of pulse intervals via ECG and the Light Heart mobile app.

### Experimental Measures

Lead II from a standard ECG sampled at 500 Hz was used to measure the R-R interval (BioAmp FE132, ADInstruments). LabChart 8 and PowerLab systems (ADInstruments) were used for data collection and storage. Pulsatile blood volume changes in the right index finger microcirculation were detected using PPG by the Light Heart mobile app (v0.0.1-alpha) leveraging the flash and camera of an iPhone X (iOS 16, Apple).

### Data Processing and Analysis

The data processing specifications were selected a priori and refined based on trial-and-error pilot testing that was conducted during app development by the Light Heart research team. Once the parameters were established, we did not make any adjustments to them throughout the conduct of this study. Light Heart collected data at 30 frames per second and produced a PPG signal by averaging the luminosity for each frame stored in a time-stamped array. PPG data were filtered using a Butterworth bandpass filter (0.75-3 Hz) and smoothed using a 5-frame smoothing window based on the convolution of a scaled window. Data were sampled using a 180 Hz cubic spline interpolation. Data processing was performed using open-source Python 3.7.6 algorithms.

Pulse intervals were extracted from the PPG signal by detecting the local maxima of the signal using a simple neighbor comparison. Local maxima greater than 30% of the mean prominence of all local maxima detected were considered valid. During pilot testing, we started with a 25% criterion as suggested by Plews et al [16] but found that accuracy was increased when we shifted to 30%. To remove any diastolic peaks that were incorrectly identified as a systolic peak, a sliding 5-peak window was applied and any local maxima less than 75% of the mean prominence within the window were discarded, with the remaining local maxima considered valid for pulse-interval analysis. Pulse intervals (ms) were computed as the time duration between valid local maxima.

To identify remaining artifacts, the pulse interval data were processed using a multistep artifact detection approach. Light Heart performs on-device, real-time signal quality analysis on windows of 200 frames in order to provide feedback to the user about the positioning of their finger. Accordingly, pulse interval data were windowed into shorter segments of 200 intervals, and low and high threshold cutoff values were set using the first and third quartile for the respective window. Any values exceeding the thresholds were removed [21]. In addition, subsequent intervals that differed by 20% were removed as they were considered physiologically implausible. Finally, any pulse interval associated with a heart rate less than 20 beats per minute (bpm) or greater than 200 bpm was removed.

ECG data were screened to ensure no ectopic beats. Local maxima of the ECG signal associated with R-waves were detected using Python open-source algorithms (Python Software Foundation) and stored with the associated time stamp. Beat-by-beat pulse interval was computed as the time between successive R-R intervals. ECG R-waves were time aligned and cross-correlated with Light Heart–derived PPG local maxima. Finally, to facilitate agreement assessment at the beat-by-beat level, the alignment of ECG pulse interval and Light Heart pulse interval data were verified by visual inspection performed by a single investigator (SRL) [16]. For both the ECG data and Light Heart PPG data, the SD of normal pulse intervals (SDNN) provided a measure of HRV.

### Statistical Analyses

Pearson tests of correlation and intraclass correlation coefficient (ICC; 2-way random effects, absolute agreement) assessed the relationship and agreement, respectively, for the study measures. Bland-Altman plots assessed agreement between Light Heart– and ECG-based measures [22]. Fixed (paired, 2-tailed *t* test) and proportional biases (Pearson correlation) were assessed. Analyses were performed using SPSS (SPSS Inc) and Prism (GraphPad). All tests were 2-tailed; α=.01. Data are presented as mean and SD unless otherwise specified.

## Results

### Comparison of Beat-by-Beat Pulse Interval Between ECG and Light Heart

**Figure 1:**
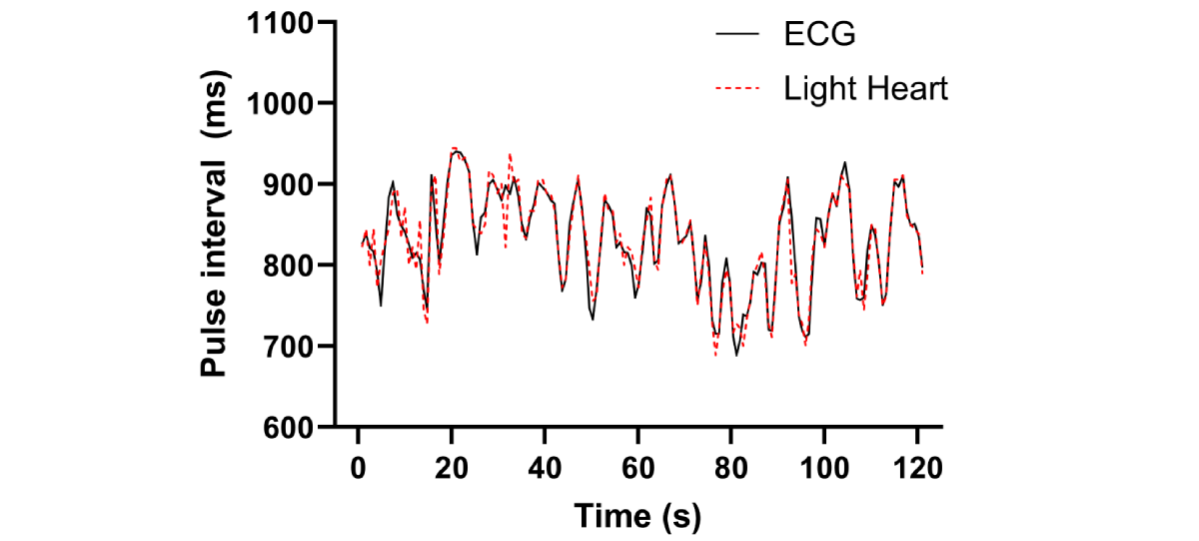
Time-aligned pulse interval data from ECG and Light Heart from 1 representative participant. ECG: electrocardiogram.

This section reports the beat-by-beat data from 5-minute pulse interval recordings in the seated posture in all individuals. Figure 1 illustrates superimposed recordings of 1 minute of pulse interval data simultaneously recorded by Light Heart and ECG, for 1 representative participant. Visual assessment of these data tracings illustrates that the pulse interval recorded by Light Heart demonstrates strong beat-to-beat agreement with the pulse interval detected by ECG. This observation was consistent across all participants. Table S1 in Multimedia Appendix 1 provides mean and variability data for pulse intervals acquired by ECG and Light Heart for all participants.

**Figure 2:**
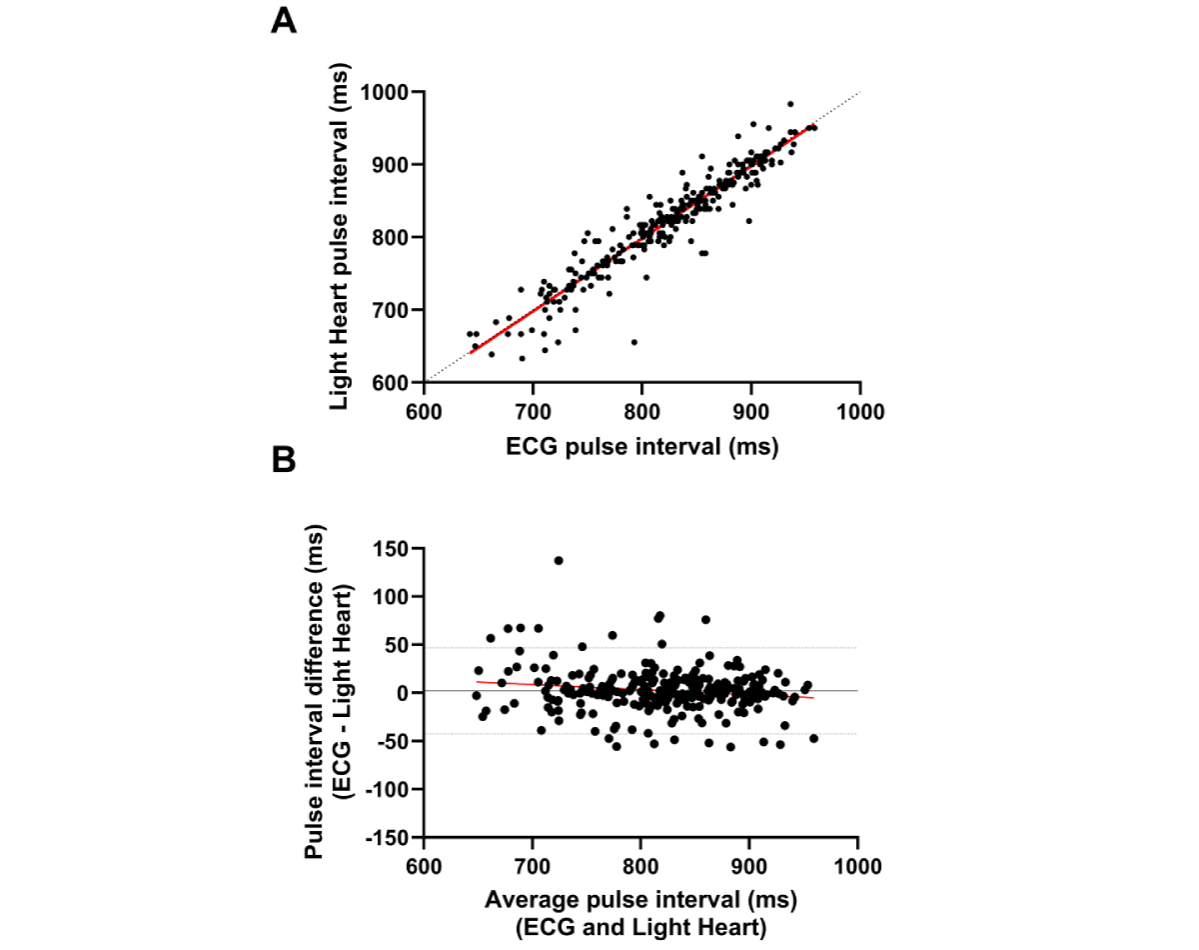
Pulse interval agreement data for the beat-by-beat analysis from 1 representative participant. The (A) scatter plot and (B) Bland-Altman plot demonstrate agreement between the pulse interval collected by ECG and the pulse interval collected by Light Heart. (A) The dashed black line represents the line of identity to assess agreement, and the solid red line represents the linear relationship between measures assessed by the Pearson correlation coefficient (*r=*0.95; *P*<.001). (B) The mean difference (2.19, SD 22.78 ms; solid black line) and 95% CIs (–42 to 47 ms; dashed black lines) are plotted. The regression line (b=–0.05±0.02; *P*=.009; R2=0.025) fitted to the difference between methods versus the mean of methods is plotted (solid red line). ECG: electrocardiogram.

Base on inspection of the beat-by-beat data in 1 representative participant (illustrated in Figure 2A), the pulse interval obtained by ECG and the pulse interval obtained by Light Heart demonstrated a strong positive correlation and agreement. Table S2 in Multimedia Appendix 1 provides the Pearson correlation coefficients and ICCs between pulse intervals collected by ECG and Light Heart for each participant. When analyses were performed on 5 minutes of beat-by-beat data, all participants demonstrated strong positive Pearson correlation coefficients ranging from 0.89 to 0.99 (*P*<.001 in 14/14, 100% participants; Table S2 in Multimedia Appendix 1). All participants demonstrated strong ICCs ranging from 0.88 to 0.99 (*P*<.001 for 14/14, 100% participants; Table S2 in Multimedia Appendix 1).

As illustrated by the Bland-Altman plot (Figure 2B) constructed with beat-by-beat data for 1 representative participant, the pulse intervals measured by ECG and Light Heart demonstrated strong agreement. For all plots, the scatter of differences was consistent over the range of mean values, and most of the differences between methods fell within the 95% CI. None of the plots demonstrated fixed bias, as small differences (range –2.9 to 2.2 ms) existed between the methods over the range of mean values and paired-sample *t* tests revealed no differences (*P*≥.11 for 14/14, 100% participants). The test of proportional bias for each participant’s beat-by-beat data, performed by regressing the difference between methods (ECG–Light Heart) against the mean of the methods, revealed proportional bias in 7 (50%) out of 14 participants (Table S3 in Multimedia Appendix 1). However, inspection of the *R*^2^ values associated with the test of proportional bias revealed demonstrably low values in all participants (*R*^2^<0.06 in 14/14, 100% participants; Table S3 in Multimedia Appendix 1), suggesting that less than 6% of the variability in the difference between methods was explained by the average value. Table S3 in Multimedia Appendix 1 provides a summary of the Bland-Altman statistics and tests of fixed and proportional bias for each participant’s beat-by-beat data.

### Comparison of Mean Pulse Interval and HRV Metrics Between ECG and Light Heart

**Figure 3:**
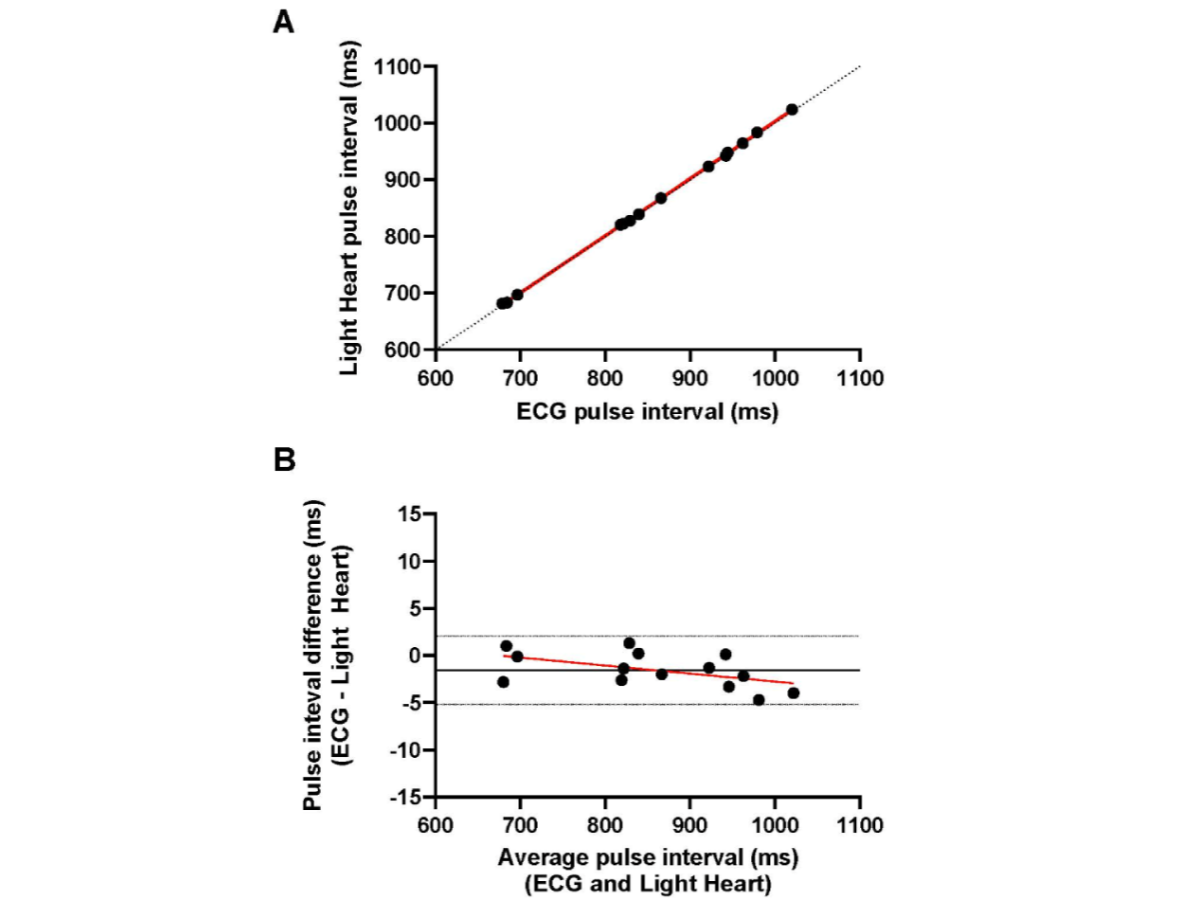
Pulse interval agreement data for the mean data analysis for the study cohort. The (A) scatter plot and (B) Bland-Altman plot demonstrate agreement between the pulse interval collected by ECG and the pulse interval collected by Light Heart. (A) The dashed black line represents the line of identity to assess agreement, and the solid red line represents the linear relationship between measures assessed by the Pearson correlation coefficient (*r=*0.99; *P*<.001). (B) The mean difference (–1.56, SD 1.86 ms; solid black line) and 95% CIs (–5.2 to 2.1 ms; dashed black lines) are plotted. The regression line (b=–0.009±0.004; *P*=.06; R2=0.27) fitted to the difference between methods versus the mean of methods is plotted (solid red line). ECG: electrocardiogram.

Inspection of the mean pulse interval data for each participant (Figure 3A) revealed that pulse intervals obtained by ECG and Light Heart demonstrated a strong positive correlation (*r*=0.99; *P*<.001) and strong agreement (ICC=1.00; 95% CI 0.99-1.00). As illustrated by the Bland-Altman plot (Figure 3B) constructed with mean data for each participant, pulse intervals measured by ECG and Light Heart demonstrated strong agreement. The scatter of differences was consistent over the range of mean values and all differences between methods fell within the 95% CI. This plot illustrated a demonstrably small but statistically significant fixed bias (–1.56, 95% CI –5.2 to 2.1 ms; *P*=.008), suggesting that Light Heart overestimated pulse interval by a small margin. The test of proportional bias performed by regressing the difference between methods (ECG–Light Heart) against the mean of the methods, revealed a trend toward proportional bias (*b*=–0.009±0.004; *P*=.06; *R*^2^=0.27).

**Figure 4:**
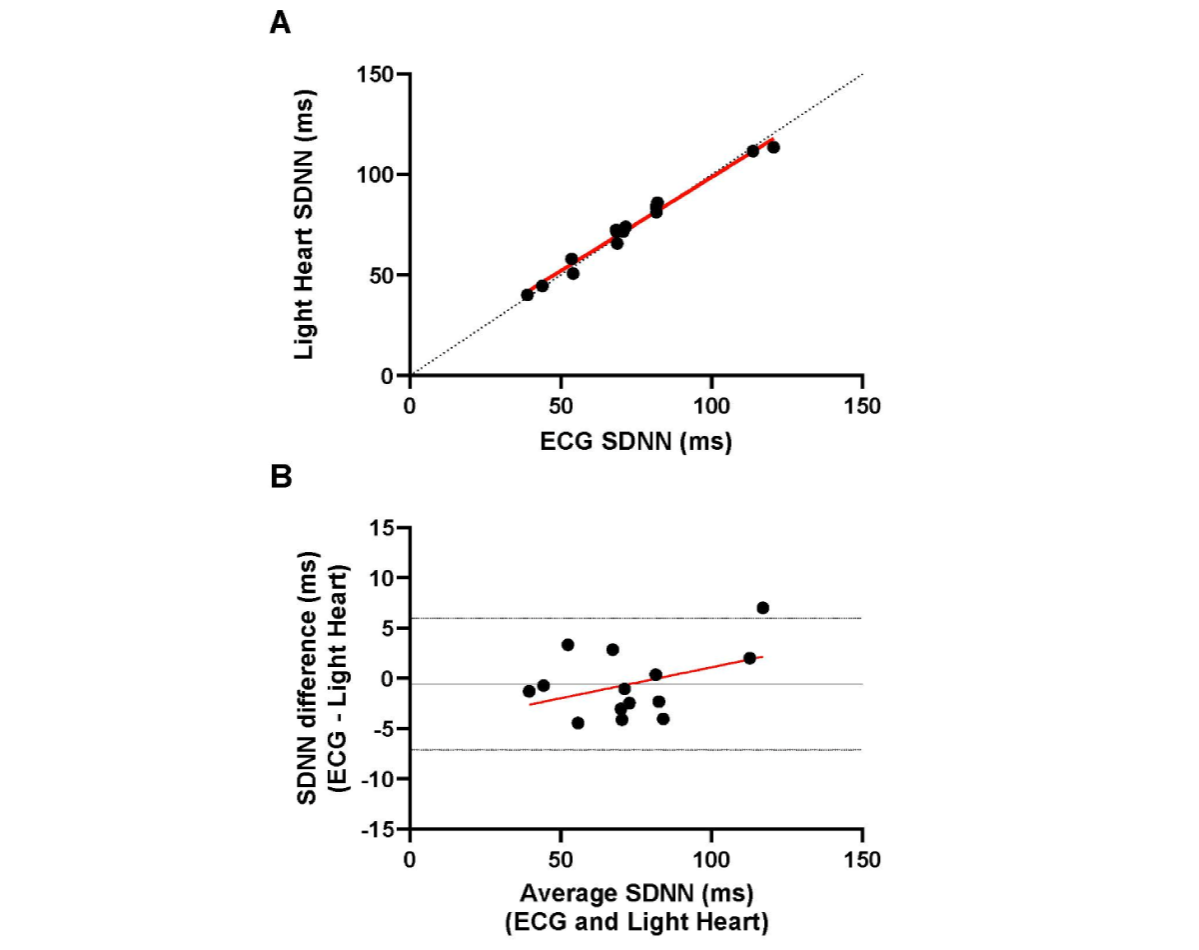
HRV agreement data for the mean data analysis for the study cohort. The (A) scatter plot and (B) Bland-Altman plot demonstrate agreement between the SDNNs collected by ECG and Light Heart. (A) The dashed black line represents the line of identity to assess agreement, and the solid red line represents the linear relationship between measures assessed by the Pearson correlation coefficient (*r=*0.99; *P*<.001). (B) The mean difference (–0.56, SD 3.34 ms; solid black line) and 95% CIs (–7.1 to 6.0 ms; dashed black lines) are plotted. The regression line (b=0.062±0.04; *P*=.14; R2=0.17) fitted to the difference between methods versus the mean of methods is plotted (solid red line). ECG: electrocardiogram; HRV: heart rate variability; SDNN: SD of normal pulse intervals.

Inspection of the mean SDNN data from each participant (Figure 4A) illustrated a strong positive correlation (*r*=0.99; *P*<.001) and strong agreement (ICC=0.99, 95% CI 0.97-1.00) between ECG and Light Heart. The Bland-Altman plot (Figure 4B) constructed with mean SDNN measured by ECG and Light Heart demonstrated strong agreement (Light Heart: mean 73, SD 23 ms; ECG: mean 73, SD 22 ms). The scatter of differences was consistent over the range of mean values, and 1 data point for the differences between the methods fell beyond the 95% CI. There was no evidence of fixed (mean difference –0.56, SD 3.34 ms; *P*=.54) or proportional bias (*b*=0.062±0.04; *P*=.14; *R*^2^=0.17).

## Discussion

### Principal Findings

This study generated novel data supporting the validity of the Light Heart mobile app’s PPG-based measures of pulse interval and HRV in a sample of healthy young individuals. The major findings of this study are as follows: (1) Light Heart–derived values of pulse interval demonstrated a strong relationship and agreement with the pulse interval measured with ECG at both the participant level and the sample level, and (2) SDNN, a measure of HRV, demonstrated a strong relationship and agreement with the SDNN calculated using ECG. These findings were based on the collective interpretations of ICCs and agreement plots generated using the technique described by Bland and Altman [22]. Therefore, Light Heart has utility as a measure of pulse interval and HRV in young healthy male and female individuals under resting seated conditions.

To our knowledge, this was the first study to assess the validity of the Light Heart mobile app’s PPG-derived indices of pulse interval and HRV. To achieve this objective, we compared the pulse interval and SDNN obtained using the Light Heart mobile app with measures collected via ECG. Three pieces of evidence suggest that Light Heart provides valid measures of pulse interval in this study. First, the pulse interval obtained from Light Heart demonstrated a strong linear relationship with the pulse interval measured via ECG. Second, the pulse interval obtained from Light Heart demonstrated a strong ICC with pulse interval measured via ECG. Third, the Bland-Altman plot demonstrated agreement between measures and included no fixed bias when beat-by-beat data were analyzed for each participant. However, fixed bias was observed when the mean pulse interval data from each participant were used to construct a Bland-Altman plot. This fixed bias suggests that Light Heart overestimates pulse interval by a demonstrably low value (<2 ms). In addition, proportional bias emerged at both the beat-by-beat analysis and mean data analysis stages, suggesting that the magnitude of pulse interval overestimation by Light Heart increases as pulse interval increases (ie, heart rate slows). Again, however, this overestimation is demonstrably low within the range of mean pulse intervals recorded in the seated posture in this study (679-1020 ms). Similarly, Light Heart provided valid measures of HRV in this study. This is not surprising given that the SDNN was calculated using pulse interval data collected by Light Heart, which aligned with ECG-derived values. This conclusion was based on the evidence of strong correlation coefficients, no fixed bias, and no proportional bias between the SDNNs measured by Light Heart and ECG.

The observation that Light Heart’s PPG-based measures of pulse interval and HRV were similar to ECG-derived measures is consistent with previous research. Indeed, Tarniceriu et al [23] found that a wrist-based plethysmographic method for pulse interval acquisition demonstrated strong agreement with R-R interval from ECG in individuals with normal sinus arrhythmia and atrial fibrillation. In addition, other groups have found good agreement in the pulse intervals between ECG and smartphone apps that use the flashlight and camera [15,16]. In our hands, our data suggest that the Light Heart app represents an additional simple and convenient PPG-based tool to add to the collection of devices that enable heart rate and HRV measures beyond research and clinical settings.

### Limitations

Several limitations of this study should be discussed. This study was performed on a sample of young healthy individuals. Thus, we are uncertain whether the validity of Light Heart is generalizable to other populations such as older individuals or patients with elevated cardiovascular risk and lower HRV [12,24,25]. The study sample included a high proportion of White individuals and was not powered to examine the validity of Light Heart in cohorts with varying skin tones. However, in 3 individuals who reported race as Southeast Asian and Hispanic or Latino, the agreement statistics discussed above were not demonstrably different than the White participants. In addition, this study was performed in a small sample of individuals (n=14). This sample size was consistent with our a priori sample size calculation and the agreement between Light Heart– and ECG-based measures was observed at both the individual and cohort levels, suggesting that the sample size likely did not influence our findings. The participants were familiarized with the Light Heart app prior to data collection, and data collection was performed during 5-minute resting seated conditions to improve the likelihood of high-quality pulse interval data collection. Future research is encouraged to replicate these findings in larger samples of diverse populations.

This study assessed the validity of R-R interval and HRV measured by the Light Heart mobile app in a sitting posture while participants were resting and breathing spontaneously. This posture aligns with instructions for how individuals perform Light Heart readings in the real world. However, study participants demonstrated a large range of resting heart rates (59-88 bpm) and proportional bias was low. This suggests that the Light Heart mobile app may demonstrate validity in other postures or conditions imposing elevated heart rates or changes in a vasomotor tone such as those experienced during daily living (eg, physical exercise and mental stress) [26]. Future research is warranted to validate the Light Heart app under various physiological conditions. Apps often take a data-driven approach during their development to maximize performance. We made adjustments to certain processing parameters used in previous research to optimize their accuracy (eg, shifting mean prominence from 25% [21] to 30% of local maxima). While all adjustments were made prior to starting this study, future research could compare the impact of these adjustments on accuracy once we obtain a larger and more diverse database. Finally, our conclusions related to the validity of Light Heart for measuring pulse interval are delimited to the mobile device and operating system used in this study.

### Conclusions and Perspectives

Through comparison with ECG, this study provides evidence to suggest that the Light Heart mobile app provides a valid measure of pulse interval and HRV in healthy young adults. HRV is strongly associated with neurological [4], metabolic [5], and cardiovascular diseases [6] and tracks positive physiological improvements with lifestyle interventions such as physical exercise [27]. Thus, combined with the high prevalence of smartphone use, our data suggest that the Light Heart app represents a simple, scalable tool that can provide insight into the human brain-heart axis and overall morbidity and mortality risk. Future research may consider incorporating Light Heart into biofeedback interventions designed to improve HRV on varying time scales.

## Appendix

Multimedia Appendix 1 Mean and variability of R-R interval and pulse interval measures by participant, Pearson correlation coefficients and intraclass correlation coefficients between pulse intervals collected by ECG and Light Heart for each participant, and Bland-Atman statistics of agreement for beat-by-beat pulse interval.

## Abbreviations

bpm: beats per minute
ECG: electrocardiogram
HRV: heart rate variability
ICC: intraclass correlation coefficient
PPG: photoplethysmography
SDNN: SD of normal pulse intervals

## Data Availability

The data sets generated during or analyzed during this study are available from the corresponding author upon reasonable request.
