# Supplementary material for: Examining the Light Heart Mobile Device App for Assessing Human Pulse Interval and Heart Rate Variability: Validation Study
Source: JMIR Form Res. 2024 Aug 20;8:e56921. doi: 10.2196/56921 (PMC11372322; doi:10.2196/56921)
Supplement: Multimedia Appendix 1 [file formative_v8i1e56921_app1.docx]

**Contents**

**Table S1Page 2**

**Table S2 Page 3**

**Table S3Page 4**

| **Table S1.** Mean and variability of R-R interval and pulse interval measures by participant. | | | | | | | | | |  |
| --- | --- | --- | --- | --- | --- | --- | --- | --- | --- | --- |
|  | **ECG Pulse Interval** | | | |  | **Light Heart Pulse Interval** | | | |  |
| **Participant** | **Mean (ms)** | **SDNN (ms)** | **CV (%)** | **Range (ms)** |  | **Mean (ms)** | **SDNN (ms)** | **CV (%)** | **Range (ms)** |  |
| 1 | 679 | 44 | 6 | 268 |  | 682 | 45 | 7 | 272 |  |
| 2 | 942 | 69 | 7 | 448 |  | 942 | 66 | 7 | 394 |  |
| 3 | 829 | 82 | 10 | 461 |  | 828 | 81 | 10 | 400 |  |
| 4 | 944 | 82 | 9 | 425 |  | 948 | 84 | 9 | 444 |  |
| 5 | 922 | 69 | 7 | 329 |  | 923 | 72 | 8 | 433 |  |
| 6 | 697 | 39 | 6 | 223 |  | 697 | 40 | 6 | 217 |  |
| 7 | 979 | 114 | 12 | 588 |  | 984 | 112 | 11 | 561 |  |
| 8 | 684 | 82 | 12 | 408 |  | 683 | 86 | 13 | 406 |  |
| 9 | 1020 | 68 | 7 | 331 |  | 1024 | 72 | 7 | 422 |  |
| 10 | 962 | 54 | 6 | 303 |  | 964 | 58 | 6 | 311 |  |
| 11 | 818 | 54 | 7 | 311 |  | 821 | 51 | 6 | 267 |  |
| 12 | 821 | 71 | 9 | 316 |  | 822 | 72 | 9 | 350 |  |
| 13 | 840 | 121 | 14 | 668 |  | 839 | 114 | 14 | 622 |  |
| 14 | 866 | 72 | 8 | 372 |  | 868 | 74 | 9 | 344 |  |
| Note: CV, coefficient of variation. SDNN, standard deviation of normal pulse intervals | | | | | | | | | |  |
|  |  |  |  |  |  |  |  |  |  |  |
|  |  |  |  |  |  |  |  |  |  |  |
|  |  |  |  |  |  |  |  |  |  |  |

| **Table S2.** Pearson correlation coefficients and intraclass correlation coefficients between pulse intervals collected by ECG and Light Heart for each participant. | | |  |
| --- | --- | --- | --- |
| **Participant** | **Pearson's *r* ^a^** | **ICC (95% CI) ^a^** |  |
| 1 | 0.93 | 0.93 (0.92, 0.95) |  |
| 2 | 0.98 | 0.98 (0.97, 0.98) |  |
| 3 | 0.99 | 0.99 (0.99,0.99) |  |
| 4 | 0.96 | 0.96 (0.95, 0.97) |  |
| 5 | 0.94 | 0.94 (0.92, 0.95) |  |
| 6 | 0.97 | 0.97 (0.96, 0.97) |  |
| 7 | 0.96 | 0.96 (0.95, 0.97) |  |
| 8 | 0.98 | 0.98 (0.97, 0.98) |  |
| 9 | 0.89 | 0.88 (0.85, 0.90) |  |
| 10 | 0.91 | 0.91 (0.88, 0.93) |  |
| 11 | 0.92 | 0.92 (0.90, 0.94) |  |
| 12 | 0.95 | 0.95 (0.93, 0.96) |  |
| 13 | 0.96 | 0.96 (0.95, 0.97) |  |
| 14 | 0.91 | 0.91 (0.89, 0.93) |  |
| Note: ^a^, denotes all *P* values associated with correlation coefficients are *P* < 0.001  95% CI, 95% confidence interval; ICC, intra-class correlation coefficient. | | |  |
|  |  |  |  |
|  |  |  |  |
|  |  |  |  |

| **Table S3.** Bland-Atman statistics of agreement for beat-by-beat pulse interval. | | | | |  |  |  |
| --- | --- | --- | --- | --- | --- | --- | --- |
|  | **Fixed Bias** | |  | **Proportional Bias** | | |  |
| **Participant** | **Mean Difference (95% CI)** | **Paired t-test *P*-value** |  | **b (SE)** | **R^2^** | **Pearson's *r P*-value** |  |
| 1 | -1.00 (-32, 30) | 0.29 |  | -0.03 (0.02) | 0.01 | 0.18 |  |
| 2 | -0.35 (-26, 27) | 0.67 |  | 0.01 (0.01) | 0.00 | 0.27 |  |
| 3 | 0.27 (-22, 22) | 0.67 |  | -0.02 (0.01) | 0.01 | 0.04 |  |
| 4 | -0.62 (-46, 45) | 0.67 |  | -0.04 (0.02) | 0.02 | 0.02 |  |
| 5 | -1.35 (-50, 47) | 0.35 |  | -0.04 (0.02) | 0.01 | 0.06 |  |
| 6 | -0.43 (-20, 20) | 0.41 |  | -0.03 (0.01) | 0.01 | 0.04 |  |
| 7 | -0.10 (-61, 61) | 0.96 |  | 0.01 (0.02) | 0.00 | 0.52 |  |
| 8 | 1.43 (-33, 36) | 0.14 |  | -0.03 (0.01) | 0.02 | 0.01 |  |
| 9 | -2.14 (-68, 64) | 0.28 |  | -0.11 (0.03) | 0.04 | <0.001 |  |
| 10 | 0.72 (-46, 47) | 0.64 |  | -0.11 (0.03) | 0.06 | < 0.0001 |  |
| 11 | 0.18 (-40, 40) | 0.88 |  | 0.00 (0.02) | 0.00 | 0.97 |  |
| 12 | 2.19 (-42, 47) | 0.11 |  | -0.05 (0.02) | 0.03 | 0.01 |  |
| 13 | -2.91 (-68, 62) | 0.16 |  | 0.00 (0.02) | 0.00 | 0.84 |  |
| 14 | -0.78 (-62, 61) | 0.68 |  | -0.03 (0.03) | 0.01 | 0.21 |  |
| Note: 95% CI, 95% confidence interval. | | | | | | |  |
|  |  |  |  |  |  |  |  |
|  |  |  |  |  |  |  |  |
|  |  |  |  |  |  |  |  |
